# Ultrafast diffusion exchange nuclear magnetic resonance

**DOI:** 10.1038/s41467-020-17079-7

**Published:** 2020-06-26

**Authors:** Otto Mankinen, Vladimir V. Zhivonitko, Anne Selent, Sarah Mailhiot, Sanna Komulainen, Nønne L. Prisle, Susanna Ahola, Ville-Veikko Telkki

**Affiliations:** 1 NMR Research Unit, University of Oulu, P.O.Box 3000, FIN-90014 Oulu, Finland; 2 NANOMO Research Unit, University of Oulu, P.O. Box 3000, FIN-90014 Oulu, Finland

**Keywords:** Solution-state NMR, Physical chemistry

## Abstract

The exchange of molecules between different physical or chemical environments due to diffusion or chemical transformations has a crucial role in a plethora of fundamental processes such as breathing, protein folding, chemical reactions and catalysis. Here, we introduce a method for a single-scan, ultrafast NMR analysis of molecular exchange based on the diffusion coefficient contrast. The method shortens the experiment time by one to four orders of magnitude. Consequently, it opens the way for high sensitivity quantification of important transient physical and chemical exchange processes such as in cellular metabolism. As a proof of principle, we demonstrate that the method reveals the structure of aggregates formed by surfactants relevant to aerosol research.

## Abstract (web summary)

Analysis of exchange processes is time consuming by two-dimensional exchange NMR spectroscopy. Here the authors demonstrate a single-scan ultrafast Laplace NMR approach based on spatial encoding to measure molecular diffusion, with an increase by a factor six in the sensitivity per unit time.

## Introduction

The direct observation of molecular exchange is challenging, because typically the processes occur in a liquid or gas within a solid matrix. Nuclear magnetic resonance (NMR) spectroscopy provides the means to study dynamics of molecules non-invasively, without tracers, and even inside optically opaque matter^1,2^. Two-dimensional (2D) exchange spectroscopy (EXSY) enables comprehensive analysis of complex exchange processes^3^. However, the 2D measurements are time consuming, as the experiment has to be repeated from tens to hundreds of times with an incremented evolution time.

Ultrafast (UF) NMR spectroscopy relies on spatial encoding of incremented evolution times into the layers of a sample by exploiting the principles of magnetic resonance imaging (MRI)^4,5^. In theory, the UF approach is capable of delivering any kind of 2D NMR spectra in a single scan^6^, and spatial encoding in discrete layers by using frequency selective pulses along with gradients have been exploited in the measurement of single-scan EXSY spectrum^7^. Furthermore, under-sampled, small flip-angle single-scan EXSY was demonstrated based on the phase accrual during echo times^8^. In those experiments, the indirect dimension was heavily under-sampled (only three points collected) and only a small part of the full magnetization was used in the detection due to the small flip angle excitation. On the other hand, the method could probe 5–20 mixing times in a single scan, leading to a comprehensive characterization of the exchange process.

Recently, we have demonstrated that the principles of spatial encoding can be extended to multidimensional relaxation and diffusion correlation experiments under the concept of ultrafast Laplace NMR (UF LNMR)^9,10^. Here, we show that UF LNMR allows also single-scan exchange measurements via a Laplace NMR contrast (in this case molecular diffusion coefficient *D*). The LNMR contrast is especially useful in the case of physical exchange, such as diffusion-driven pore-to-pore exchange of fluid molecules in porous materials^11^ or intracellular to extracellular exchange of metabolites in cancer cell suspension^12^, when the exchanging sites are not resolved in spectrum. On the other hand, the LNMR contrast can be exploited in the investigation of chemical exchange as well, if the chemical interaction changes the diffusion coefficient (or relaxation time) of the spins. Contrary to the single-scan EXSY methods described above, the method introduced here is based on continuous spatial encoding and non-under-sampled data.

## Results

### Ultrafast diffusion exchange experiment

**Fig. 1:**
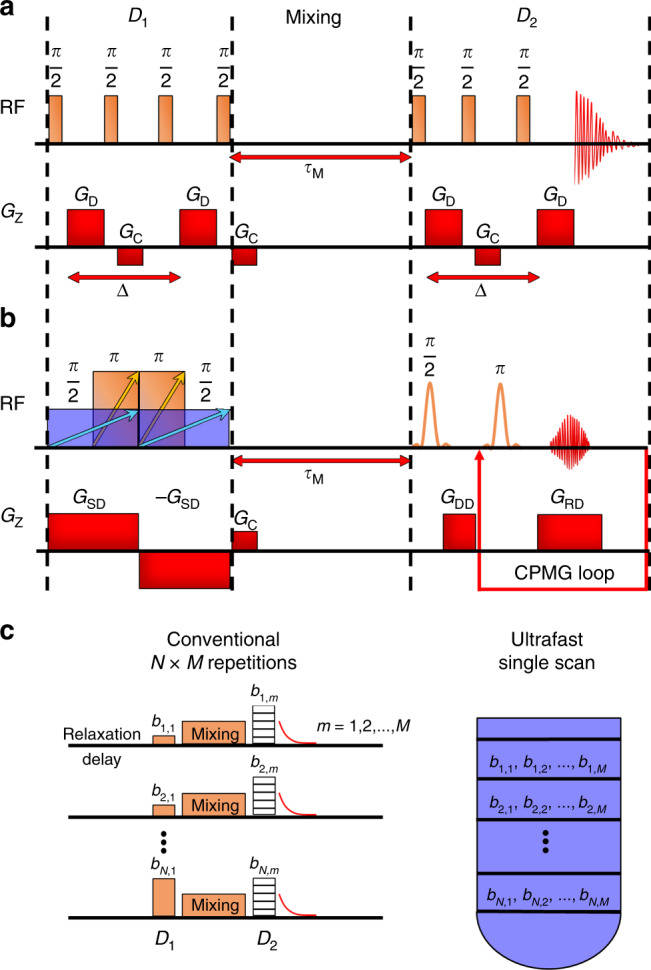
Diffusion NMR based measurements of molecular exchange. The pulse sequences for (**a**) conventional and (**b**) ultrafast diffusion exchange spectroscopy (DEXSY) measurements. The conventional experiment is repeated multiple times with varying diffusion gradient strength *G*_D_ (changed independently for periods *D*_1_ and *D*_2_), while the same data is measured in a single scan by the ultrafast approach. *D*_1_ and *D*_2_ refer to the first and second diffusion encoding periods, *τ*_M_ to the mixing time, RF to the radio frequency pulse, *G* to the gradient pulse, and Δ to the diffusion delay. Subscripts D, C, SD, DD, and RD of *G* stand for diffusion, crusher, spatial and diffusion encoding, diffusion and dephasing, and read and diffusion, respectively. The arrows overlaying the RF pulses show the direction of frequency sweep. **c** Illustration of the main difference between the conventional and ultrafast approach. In the conventional experiment, *N* and *M* data points, corresponding to the indirect and direct dimension (*D*_1_ and *D*_2_) and characterized by different *b* values (which are proportional to the gradient strength), are collected in separate experiments, leading to *N* × *M* repetitions. In the ultrafast experiment, the same data is spatially encoded into the layers of the sample in a single scan.

The pulse sequence for the conventional diffusion exchange spectroscopy (DEXSY) experiment (Fig. 1a)^13^ includes two diffusion-encoding blocks separated by the mixing time *τ*_M_. The experiment correlates the initial and final diffusion coefficients. The change in *D* indicates that the molecules have moved to a different physical or chemical environment during the mixing period. The conventional DEXSY experiment is extremely slow, because each point corresponding to both first and second diffusion encoding has to be collected in a separate experiment. As illustrated in Fig. 1c, if the numbers of points collected in the indirect and direct dimensions are *N* and *M*, the experiment requires *N* × *M* repetitions. For example, in order to collect 50 data points in each direction, the experiment has to be repeated at least 50^2^ = 2500 times (multiplied by potential additional scans for signal averaging and phase cycling). For typical samples, the repetition time (including the relaxation delay) of the experiment varies from 5 to 60 s, leading to a one scan experiment time of 3.5–42 h.

Our UF counterpart of the DEXSY experiment is shown in Fig. 1b. The first diffusion-encoding block before *τ*_M_ includes simultaneous frequency-swept and gradient pulses. The frequency of the former pulses is linearly increasing with time, while the gradient pulses make the Larmor frequency of nuclei linearly dependent on position. Therefore, the initial π/2 pulse excites the spins in the bottom of the sample tube first and those in the top last. The first π pulse is switched on at the midpoint of the π/2 pulse, and its length is a half of the π/2 pulse. The spins in different layers form an echo at the midpoint of the first diffusion-encoding block, and the echo time is linearly dependent on position (zero in the top and equal to the length of the π/2 pulse in the bottom). However, no collective echo is observed due to the different phases of the frequency-swept pulses experienced by the spins in different layers^14^. The quadratic spatial dependence of the phases is compensated out by the second set of identical frequency-swept pulses arranged symmetrically around the midpoint of the encoding block, along with another gradient pulse with an opposite polarity. Overall, the first encoding block consists of a double spin echo with the total echo time linearly dependent on the position. If the gradients are strong enough, the decay of the signal is dominated by diffusion instead of *T*_2_ relaxation. Therefore, the longitudinal magnetization profile represents the diffusion decay curve (see “Methods” section).

After the mixing period, the magnetization profile is read by applying the principles of MRI, i.e., using read gradients within a CPMG^15^ loop. Due to the presence of the strong gradients, the signal decay in the CPMG loop is also dominated by diffusion instead of *T*_2_ relaxation (see “Methods” section). Therefore, the data equivalent to conventional DEXSY experiment is measured by a single-scan, whereas, as explained above, the conventional experiment requires hundreds to thousands of repetitions. The UF approach introduced here accelerates the DEXSY experiment in two groundbreaking ways: first, the data corresponding to the indirect dimension is probed in a single scan based on spatial encoding; second, contrary to the conventional DEXSY^13^, the direct dimension is detected in a single scan based on CPMG accompanied with gradients. In some studies, CPMG sequence has been used to detect diffusion in constant field gradient experiments^16^, to accelerate pulsed-field-gradient diffusion experiments^17^ and to remove effects of field-inhomogeneity and exchange^18^, but it has not been used in DEXSY experiments or LNMR experiments aiming at determination of distributions of diffusion coefficients by the Laplace inversion. The drawback of the UF DEXSY experiment is that the spectral resolution is lost due to the spatial encoding. A partial spectral resolution can be achieved by exciting one peak from the spectrum by using frequency selective pulses in the CPMG loop, as was done in this work. On the other hand, quite often in the case of physical exchange the resonances of exchanging sites are overlapping, and spectral resolution does not provide any additional information.

### Exchange through a lipid bilayer

To demonstrate the feasibility and utility of the UF DEXSY based molecular exchange experiment, we studied the exchange of water in an aqueous sodium decanoate surfactant system. Surfactants are a class of chemicals that lowers the surface tension of liquids. They are widely exploited in industrial applications as detergents, wetting agents, foaming agents, emulsifiers etc^19^. They have also significant effect on the properties of aerosols, which are key components of the climate system^20,21^. Decanoic acid is one of the amphiphiles found from carbonaceous meteorites^22^. Our sample included 717 mM of sodium decanoate in water (10% H_2_O and 90% D_2_O).

**Fig. 2:**
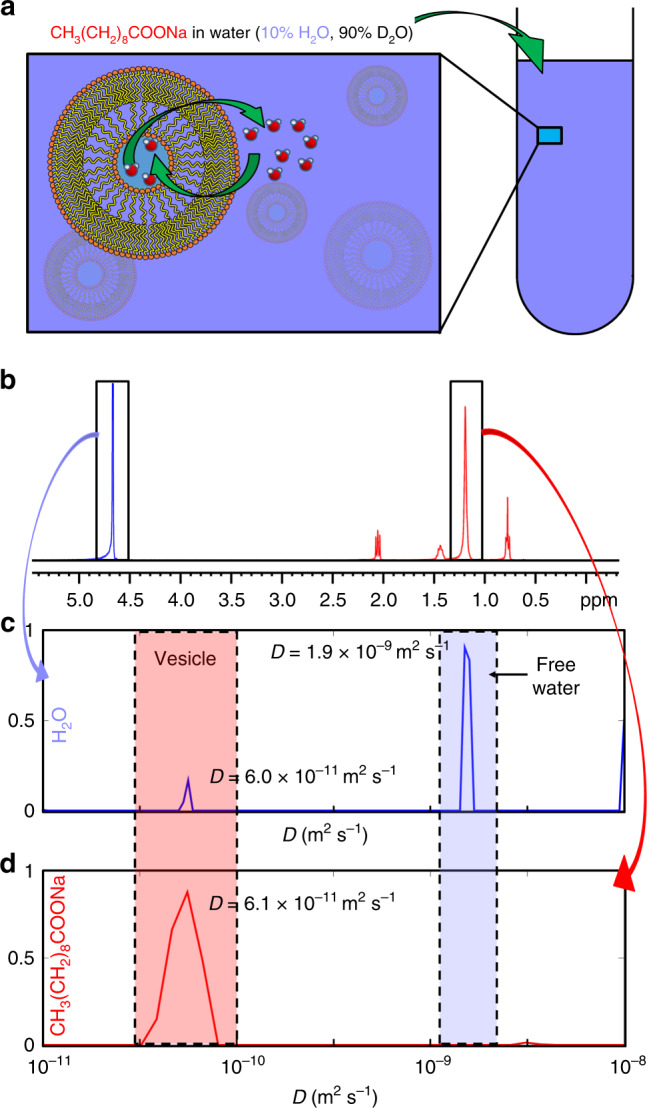
Aqueous sodium decanoate (717 mM) sample. **a** UF DEXSY analysis implies that decanoate molecules, illustrated by an orange hydrophilic head and a yellow hydrophobic tail, form vesicles and that there is a diffusion driven exchange between the water molecules (oxygens and hydrogens illustrated by red and white) encapsulated within vesicles and free water molecules. Green arrows represent the exchange process. **b** ^1^H spectrum of the sample. Blue and red colors highlight the water and decanoid signals, respectively. **c** Diffusion coefficient distribution of water measured by diffusion-ordered spectroscopy (DOSY). **d** DOSY diffusion coefficient distribution of decanoate. Blue and red arrows indicate, from which signals in the spectrum the diffusion coefficient distributions were extracted. Shaded red and blue areas indicate the diffusion coefficient regions of vesicles and free water, respectively.

^1^H NMR experiments were performed using a 400 MHz spectrometer at room temperature. Conventional diffusion-ordered spectroscopy (DOSY)^23^ analysis revealed a bimodal diffusion coefficient distribution of water molecules with significantly different *D* values, 6.0 × 10^−11^ and 1.9 × 10^−9^ m^2^ s^−1^ (Fig. 2b). The higher value corresponds to the *D* of free water, whereas the lower *D* value is equal to the *D* of decanoate (Fig. 2b). The results indicate that decanoate molecules form vesicles (Fig. 2a)^24^ and some water molecules are encapsulated inside the vesicles. The encapsulated water molecules diffuse at the same rate as the vesicles. According to the Stokes-Einstein equation^25^ and the *D* of decanoate, the diameter of the spherical vesicles is about 6.4 nm. Because the length of the decanoate molecule is about 1.2 nm, the diameter of the water capsule inside the vesicle is about 1.6 nm.

**Fig. 3:**
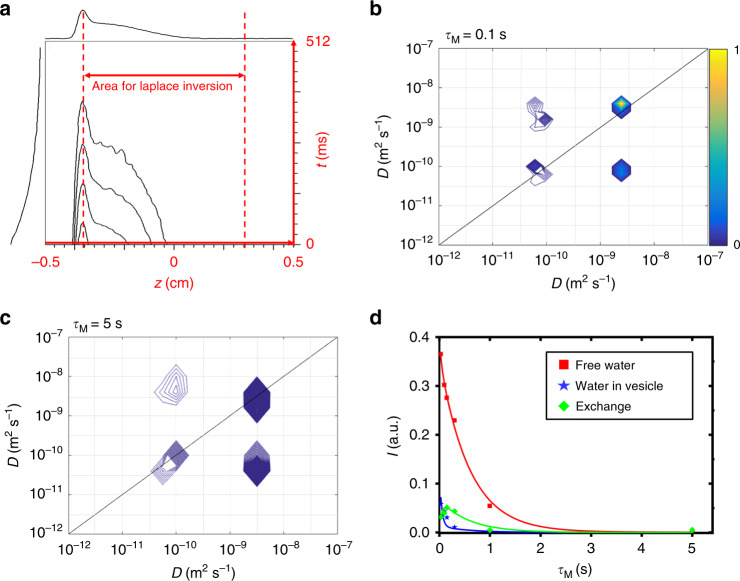
Ultrafast DEXSY analysis of molecular exchange. **a** Experimental data after the Fourier transform along the spatial encoding direction measured with a mixing time,*τ*_M_, of 1 s. The first row and the first column, which was selected for Laplace inversion, are shown on top and left, respectively. **b**, **c** Diffusion exchange maps resulting from 2D Laplace in version of the DEXSY data. **d** Integrals of the peaks in the diffusion exchange maps and the fit of two site exchange model to the data points. The fit resulted in the exchange rate value of *k* = 28 ± 6 s^−1^.

The data from a UF DEXSY measurement after the Fourier transform of the spatial encoding dimension is shown in Fig. 3a. The signal of water was exclusively selected by using frequency selective pulses in the CPMG loop (Fig. 1b). A row and a column of the 2D data shown on the top and left of the figure shows clearly the existence of two diffusion components decaying with different rates; the rapidly decaying part corresponds to the fast diffusing free water and the slowly decaying part represents the encapsulated water. The data processing is explained in detail in “Methods” section.

The 2D diffusion exchange maps resulting from 2D Laplace inversions^26,27^ are shown in Fig. 3b, c (see also Supplementary Fig. 1). The maps include two diagonal peaks corresponding to water molecules located in the same site before and after the mixing time, as well as two off-diagonal cross peaks with their coordinates corresponding to *D* of free water in the first dimension and that of encapsulated water in the second dimension or vice versa. The cross peaks reveal unambiguously the exchange of water molecules between the two sites due to diffusion through the decanoate bilayer^28^. The intensity of the cross-peaks increases first with mixing time due to increased number of water molecules passing through the bilayer and later it is decreasing due to effect of *T*_1_ relaxation (Fig. 3d).

To quantify the exchange rates, the integrals of the diagonal and cross peaks were fit with a two-site exchange model^3^ (Fig. 3d). The exchange rates (*k* = 28 ± 6 s^−1^, *k*_FE_ = 21 ± 5 s^−1^, *k*_EF_ = 7.0 ± 1.1 s^−1^) are in good agreement with the conventional DEXSY analysis (see “Methods” section and Supplementary Fig. 2). However, the experiment time of a single conventional DEXSY experiment with 8 scans and 16 diffusion encoding gradient steps in each dimension was 34 h, while the UF DEXSY measurement with 64 scans took only 1 h (the repetition time in both experiments was long, 60 s, because *T*_1_ of H_2_O was 11 s). The overall duration of the exchange experiments comprising 6 different mixing times was decreased from 9 days to 7 h by switching from the conventional to UF approach. This is a very significant reduction of the instrument time and it reduces substantially the possibilities for sample degradation during the experiments. Furthermore, much higher number of points were collected in the UF (71 and 32 points in the indirect and direct dimensions) than in the conventional experiments (16 points in the both dimensions), which led significantly better resolution in the UF DEXSY maps (cf. Fig. 3 and Supplementary Figs. 1 and 2).

## Discussion

A corresponding single-scan UF DEXSY measurement (including the relaxation delay) takes only 1 min, while a one-scan conventional experiment with the same resolution (71 × 32 data points) is 2272-times longer (38 h). We estimated that the spatial encoding lowers the sensitivity of the single-scan UF experiment by the factor of 9 as compared to the one-scan conventional experiment. However, the sensitivity per unit time (i.e., SNR in the experiment with the experiment time equal to that of the conventional experiment) is increased by the factor of 6, because the UF DEXSY experiment can be repeated 2272 times during a single conventional DEXSY experiment. We note that replacing the PGSE or PGSTE type second diffusion encoding block in the conventional DEXSY experiment by the CPMG block in the UF DEXSY experiment does not lower the sensitivity of the experiment, as full magnetization is detected in the CPMG loop. However, it has some consequences on the resolution, as the maximum resolutions in the indirect and direct dimensions are coupled, i.e., one cannot be increased without decreasing another (see “Methods” section, subsection Resolution in UF DEXSY experiments). Anyway, sufficient resolution can be achieved in both dimensions in typical liquid state studies, but the resolution may be a limiting factor in gas phase studies.

The spatial encoding block used in the UF DEXSY experiment shown in Fig. 1b differs from the spatial encoding blocks used in earlier diffusion studies^10,29–31^. In the earlier studies, the spatial encoding was based on the modified PGSE and PGSTE sequences. Here, it is based on the double spin-echo sequence, in which the gradient is on for the whole sequence. Consequently, the spatial encoding of diffusion requires less time than in the case of PGSE and PGSTE based sequences, in which the pulsed gradients are switched on only for a short time. Another benefit of the current UF DEXSY sequence is that it can serve also as UF *T*_2_–*T*_2_ exchange sequence, if the gradients are small enough so that *T*_2_ decay dominates over *D* decay (see “Methods” section). On the other hand, the spatial encoding block used in this work can be replaced by any other spatial diffusion encoding block supplemented with a π/2 pulse for storing magnetization along longitudinal direction for the mixing time. The PGSE and PGSTE based spatial diffusion encoding blocks do not require frequency-swept π/2 pulses, which are challenging to calibrate, and they do not include two simultaneous frequency-swept pulses. Therefore, they may be more robust alternatives in many applications. For the completeness, we note also that, in addition to the spatial encoding, there are many other approaches for accelerating NMR diffusion measurements, which are based on small flip angle pulses and trains of diffusion gradients, as well as non-uniform sampling^32–37^.

The UF approach has the potential to exploit modern nuclear spin hyperpolarization techniques^38,39^ to boost the sensitivity of the DEXSY experiment by several orders of magnitude, because the whole 2D data can be measured in a single scan after the hyperpolarization process^10^. This makes much smaller amounts of substances (even physiological concentrations)^40^ observable. In the conventional DEXSY experiment, the hyperpolarization process should be repeated before each repetition of the experiment, altogether from hundreds to thousands of times. In practise, this is not feasible because, for example, the build-up of dynamic nuclear polarization (DNP) takes from tens of minutes to hours^12^. Therefore, the UF approach has a potential to improve simultaneously both the time efficiency and sensitivity of molecular exchange analysis by several orders of magnitude. Combining UF DEXSY with hyperpolarization should be feasible, as hyperpolarization has been successfully exploited in other kinds of UF experiments^8,10,12,41^, even with gases^42^.

The high efficiency and sensitivity of the UF DEXSY can be utilized in a variety of important molecular exchange processes in chemistry, biochemistry and medicine, including those in cancer cells^12^, exosomes, aerosols, catalysts, porous media etc. The method is non-invasive and it does not require the use of tracers. It provides information, which is challenging to reach with any other methods. For example, the conventional EXSY cannot probe the water exchange phenomena described in this communication, because the exchange sites are not resolved in an NMR spectrum, and the standard optical methods, such as nanoparticle tracking analysis^43^, cannot detect such small (6.4 nm) vesicles, which were observed in the UF DEXSY analysis. It is possible to switch from diffusion to *T*_2_ relaxation contrast in the exchange analysis, if small gradients are used in the UF measurements (see “Methods” section), in which case the contrast reflects predominantly rotational motion of the molecules instead of translational^44^. The time window of molecular processes observable by the UF DEXSY can be extended at least by an order of magnitude by exploiting long-lived states^45,46^, enabling the investigation of slow exchange processes. Because the UF LNMR experiments are based on spin echoes, they are applicable even with low-field, portable and affordable NMR devices^47^ with inhomogeneous magnetic fields^48,49^. Therefore, hyperpolarized, mobile UF LNMR has the potential to bring advanced, high sensitivity and cost-efficient analysis of molecular exchange processes outside laboratories.

## Methods

### Experimental

The sample included 717 mM sodium decanoate (Sigma-Aldrich, purity ≥98%) in water (90% D_2_O, 10% H_2_O) in a sealed 5 mm NMR tube. The pH of the sample was about 8. NMR experiments were carried out on a Bruker Avance III 400 MHz spectrometer equipped with 5 mm BBO probe at room temperature.

Conventional DEXSY experiments: The number of diffusion gradient steps was 16 in both dimensions and the amplitudes of the gradients were linearly increased from 26.7 to 508 mT m^−1^. The length of the diffusion gradients (*δ*) was 2.6 ms and the diffusion delay (∆) was 100 ms. The number of accumulated scans was 8, the relaxation delay was 60 s and the experiment time was 34 h. Altogether 6 experiments were performed with the mixing time varying from 5 ms to 5 s.

Ultrafast DEXSY experiments: The lengths of 90° and 180° frequency-swept pulses were 15 and 7.5 ms, respectively, and the sweeping width Δ*ν* was 144 kHz. The amplitude of the spatial/diffusion encoding gradient *G*_SD_ was 508 mT m^−1^. The number of echoes in the CPMG loop was 32, and the echo time was 8 ms. The number of complex data points collected for each echo was 128, dwell time was 5 μs and the acquisition time of an echo was 1.28 ms. The amplitude of the trapezoidal read gradient *G*_RD_ was 268 mT m^−1^, the gradient ramp time was 0.5 ms and the overall length of the gradient was 3.94 ms (including the ramping times). The length of Gaussian frequency selective 90° and 180° in the CPMG loop was 1 ms. The number of accumulated scans was 64, the relaxation delay was 60 s and the experiment time was 1 h 5 min. Altogether 6 experiments were performed with the mixing time *τ*_M_ varying from 30 ms to 5 s. There are small artefacts at the edges and in the center of the spatial encoding profile of the UF DEXSY experiment due to imperfect initial/final performance of the frequency-swept pulses (see Supplementary Fig. 3). Before the 2D Laplace inversion, the data points suffering from the artefacts, as well as the data outside the region affected by the frequency-swept inversion pulse were removed, and the *z* axis was converted into the *t*_1_ axis using Eq. (7) shown below.

2D Laplace inversions were performed using the Iterative Thresholding Algorithm for Multi-exponential Decay (ITAMeD)^27^.

### Theoretical background of the UF DEXSY experiment

The frequency *ν*_F_ of the frequency-swept pulse is linearly increasing with time *t*:

1 $$\nu _{\mathrm{F}} = \frac{{\Delta \nu }}{{t_{\mathrm{F}}}}t,\,{\mathrm{when}}\, - \frac{{t_{\mathrm{F}}}}{2} \le t \le \frac{{t_{\mathrm{F}}}}{2}.$$

Here, Δ*ν* is the sweep width and *t*_F_ is the length of the pulse. The phase of the frequency-swept pulse, *ϕ*_F_, is quadratically dependent on time:

2 $$\phi _{\mathrm{F}} = \phi _{\mathrm{F}}^0 + {\int} 2 \pi \nu _{\mathrm{F}}{\mathrm{d}}t = \phi _{\mathrm{F}}^0 + \frac{{\pi \Delta \nu }}{{t_{\mathrm{F}}}}t^2.$$

Here, $\phi _{\mathrm{F}}^0$ is the phase at *t* = 0. In the presence of the spatial/diffusion encoding gradient *G*_SD_, the Larmor frequency of the nuclei, *ν*_L_, is linearly dependent on position, *z*:

3 $$\nu _{\mathrm{L}} = \frac{{\gamma G_{{\mathrm{SD}}}}}{{2\pi }}z.$$

Here, *γ* is the gyromagnetic ratio of the nuclei. The spins at position *z* are excited/inverted when $\nu _{\mathrm{F}} = \nu _{\mathrm{L}}$ (the validity of the instantaneous excitation/inversion approximation is discussed in ref. ^50^), and, according to Eqs. (1) and (3), the excitation/inversion time instant of the spins is linearly dependent on position:

4 $$t\left( z \right) = \frac{{\gamma G_{{\mathrm{SD}}}t_{\mathrm{F}}}}{{2\pi \Delta \nu }}z.$$

Because the minimum and maximum frequencies of the frequency-swept pulse are $- \frac{{\Delta \nu }}{2}$ and $+ \frac{{\Delta \nu }}{2}$, the maximum and minimum positions affected by the pulse are

5 $$z_{{\mathrm{max}}/{\mathrm{min}}} = \pm \frac{{\pi {\mathrm{\Delta }}\nu }}{{\gamma G_{{\mathrm{SD}}}}}.$$

According to Eqs. (2) and (4), the phase of the frequency-swept pulse experienced by spins at *z*, $\phi _{\mathrm{F}}\left( z \right)$, is quadratically dependent on position:

6 $$\phi _{\mathrm{F}}\left( z \right) = \phi _{\mathrm{F}}^0 + \frac{{\gamma ^2G_{{\mathrm{SD}}}^2t_{\mathrm{F}}}}{{4\pi \Delta \nu }}z^2.$$

In the UF DEXSY experiment, the initial π/2 frequency-swept pulse excites the spins in the bottom of the spatial encoding region right in the beginning of the pulse and those in the top at the end of the pulse. In between these extremes, the excitation time instant is linearly dependent on position (see Eq. 4). The first frequency-swept π pulse is switched on in the midpoint of the initial π/2 pulse, and its length, $t_{\mathrm{F}}^\pi$, is half of the length of the π/2 pulse, $t_{\mathrm{F}}^{\pi /2}$. As it is sweeping the same frequency range Δ*ν*, the sweep rate of the π pulse is double as compared to the π/2 pulse. Therefore, the π pulse affects the spins always in the halfway between the excitation and the end of the initial π/2 pulse. The second frequency-swept π/2 and π pulses are switched on simultaneously, and they are identical to the first pulses. However, the gradient has an opposite polarity during those pulses. Consequently, the pulses affect the spins in the top first and those in the bottom last (see Eq. 4). Overall, the first diffusion encoding period comprises a double spin echo with the total echo time linearly varying with position (zero in the top, $2t_{\mathrm{F}}^{\pi /2}$ in the bottom), and the second π/2 rotates magnetization along the longitudinal direction for the mixing time *τ*_M_.

As the phase of the first π/2 pulse, $\phi _{\mathrm{F}}^{\pi /2}(z)$, is quadratically dependent on position (see Eq. 6), it is inducing a quadratic dependence of the phase of the spins on the position *z*. However, this is compensated out by the second π/2 pulse with the same phase dependence (the phase is dependent on $G_{{\mathrm{SD}}}^2$, and therefore the change in polarity of the gradient does not change the phase). Similarly, the first π pulse is inducing its own spatial phase dependence (note that $\phi _{\mathrm{F}}^{\pi /2}(z) \ne \phi _{\mathrm{F}}^\pi (z)$, because $t_{\mathrm{F}}^{\pi /2} = 2t_{\mathrm{F}}^\pi$), which is compensated out by the second π pulse. The overall double spin echo time, *t*_1_, as a function of the position *z* is

7 $$t_1\left( z \right) = 2\left( {1 - \frac{{\gamma G_{{\mathrm{SD}}}}}{{\pi {\mathrm{\Delta }}\nu }}z} \right)t_{\mathrm{F}}^{\pi /2},{\mathrm{when}} - \frac{{\pi \Delta \nu }}{{\gamma G_{{\mathrm{SD}}}}} \le z \le + \frac{{\pi \Delta \nu }}{{\gamma G_{{\mathrm{SD}}}}}.$$

The amplitude of the echo is^50,51^

8 $$E_1\left( z \right) = E_1^0\exp \left[ { - \left( {\frac{1}{{T_2}} + \frac{{\gamma ^2G_{{\mathrm{SD}}}^2\left[ {t_1(z)} \right]^2}}{{48}}D_1} \right)t_1\left( z \right)} \right],$$

where $E_1^0$ is the initial signal amplitude and *D*_1_ is the diffusion coefficient during the first diffusion encoding block. The first term in the parentheses accounts for the signal decay due to *T*_2_ relaxation while the second term represents decay due to molecular diffusion in the presence of gradient *G*_SD_.

The echo amplitude in the CPMG loop in the second diffusion encoding block is^50,51^

9 $$E_2\left( {t_2} \right) = E_0^2\exp \left[ { - \left( {\frac{1}{{T_2}} + \frac{{\gamma ^2G_{{\mathrm{Reff}}}^2\tau ^2}}{3}D_2} \right)t_2} \right],$$

where *τ* is the time between the π/2 and π pulses and *t*_2_ is the time variable of the second dimension. *G*_Reff_ is the amplitude of a gradient pulse with the length of *τ* and its area equal to the that of the read gradient (*G*_R_) pulse. Strictly speaking, Eq. (9) is valid for a constant gradient, but, here, the read gradient was switched off for the RF pulses. As a good approximation, *G*_Reff_ was used in Eq. (9) instead of *G*_*R*_.

In the UF DEXSY experiments, the diffusion decay dominated over the *T*_2_ decay due to the use of strong gradients. Therefore, the *T*_2_ terms in Eqs. (8) and (9) can be neglected. Furthermore, there was a distribution of diffusion coefficients in the sample instead of a single *D*. Consequently, the overall signal amplitude observed in the DEXSY experiment is

10 $$E\left[ {t_1\left( z \right),t_2} \right] = 	\, E_0{\int} P \left( {D_1,D_2} \right)\exp \left[ { - \frac{{\gamma ^2G_{{\mathrm{SD}}}^2\left[ {t_1\left( z \right)} \right]^2}}{{48}}D_1t_1\left( z \right)} \right]\\ 	\exp \left[ { - \frac{{\gamma ^2G_{{\mathrm{Reff}}}^2\tau ^2}}{3}D_2t} \right]{\mathrm{d}}D_1{\mathrm{d}}D_2.$$

The 2D distribution of diffusion coefficients, *P*(*D*_1_, *D*_2_), was solved by a Laplace inversion based on non-negativity constraint and Tikhonov regularization with the *l*_1_-norm penalty function^27^.

### Exchange rates

The exchange rates were obtained by fitting a two-site exchange model into amplitudes of DEXSY peaks (see Fig. 3d and Supplementary Fig. 1)^3^. In the fits, *T*_1_ of free water was fixed to be 10.86 s, which is the value determined by an inversion recovery experiment. In the case of UF DEXSY, the observed exchange rate was *k* = 28 ±  6 s^−1^ (*k*_EF_ = 21 ± 5 s^−1^, *k*_FE_ = 7.0 ± 1.1 s^−1^), the relative populations of the free and encapsulated water pools were *x*_*F*_ = 0.76 ± 0.02 and *x*_*E*_ = 0.24 ± 0.02, and the relaxation rate in the encapsulated pool *R*_1E_ = 1/*T*_1E_ = 8.5 ± 1.1 s^−1^. In the case of conventional DEXSY, corresponding values were *k* = 32 ± 6 s^−1^ (*k*_EF_ = 22 ± 4 s^−1^, *k*_FE_ = 10 ± 2 s^−1^), *x*_F_ = 0.70 ± 0.04, *x*_E_ = 0.30 ± 0.03, and *R*_1E_ = 1/*T*_1E_ = 7.1 ± 0.8 s^−1^. The exchange rates are in good agreement within the experimental error. There are slight deviations in the populations and relaxation rates in the encapsulated pool, most probably because of much smaller amount of points collected in the conventional experiment and, consequently, different kind of probing of especially the initial part of the signal decay.

### Alternative analysis of the conventional DEXSY data

In the analysis of the conventional and UF DEXSY data described above, we did not take into account the exchange during the diffusion encoding periods. To check that this does not lead to significant errors in the resulting exchange rate values, we performed an alternative analysis of the DEXSY data measured with the mixing time of 1 s, using a two-site exchange model taking into account the exchange during the diffusion period^52–55^. According to the model, the observed signal is

11 $$S\left( {b_1,b_2,\Delta ,\tau _{\mathrm{M}}} \right) = S_0\left[ {\left. {\exp \left( { - b_1{\boldsymbol{D}}} \right)\exp \left( { - {\boldsymbol{K}}} \right)\exp \left( { - \tau _{\mathrm{M}}[{\boldsymbol{R}}_1 + {\boldsymbol{K}}]} \right)\exp \left( { - b_2{\boldsymbol{D}}} \right)\exp \left( { - {\boldsymbol{K}}} \right)} \right){\boldsymbol{M}}} \right]$$

Here, $b_i = \Delta \gamma ^2G_{\mathrm{D}}^2\delta _i^2$, where *δ*_*i*_ is the length of the gradient pulse, *i* = 1, 2 and Δ is the diffusion delay, ***D*** is the diffusion matrix

12 $${\boldsymbol{D}} = \left[ {\begin{array}{*{20}{c}} {D_{\mathrm{A}}} & 0 \\ 0 & {D_{\mathrm{B}}} \end{array}} \right],$$

***K*** is the exchange matrix

13 $${\boldsymbol{K}} = \left[ {\begin{array}{*{20}{c}} {k_{\mathrm{A}}} & { - k_{\mathrm{B}}} \\ { - k_{\mathrm{A}}} & {k_{\mathrm{B}}} \end{array}} \right],$$

***R***_1_ is the longitudinal relaxation matrix

14 $${\boldsymbol{R}}_1 = \left[ {\begin{array}{*{20}{c}} {R_{1,{\mathrm{A}}}} & 0 \\ 0 & {R_{1,{\mathrm{B}}}} \end{array}} \right],$$

and ***M*** is the magnetization vector magnetization vector (*M*_*A*_ + *M*_B_ = 1)

15 $${\boldsymbol{M}} = [M_{\mathrm{A}}M_{\mathrm{B}}]^{\mathrm{T}}.$$

Using the conservation of mass, *k*_A_ = *k*_B_*M*_B_/*M*_A_.

The fit of Eq. (11) with the DEXSY data resulted in the following parameter values: *k*_EF_ = 19.5 ± 0.3 s^−1^, *k*_FE _= 8.4 ± 0.2 s^−1^, *x*_F_ = 0.70 ± 0.01, *x*_E_ = 0.30 ± 0.01, *D*_E_ = (5.3 ± 0.2) × 10^−11^ m^2^ s^−1^, *D*_F_ = (1.49 ± 0.03) × 10^−9^ m^2^ s^−1^, *T*_1E_ = 9.98 ± 0.03 s and *T*_1F_ = 9.96 ± 0.03 s. The exchange rates are in agreement with the previous analysis within the error limits, confirming that it is reasonably good approximation to neglect exchange during the diffusion encoding. Other parameters are also in good agreement with the previous parameters.

### Resolution in UF DEXSY experiments

The strength (or length) of the spatial and diffusion encoding gradient *G*_SD_ has to be adjusted so that the decay of the transverse magnetization is sufficient, i.e., the smallest value of the transverse magnetization is about 5–10% of the highest value (see Supplementary Fig. 3). The smaller *D*, the higher *G*_SD_. After setting an appropriate *G*_SD_, the sweeping width of the frequency-swept pulses has to be adjusted so that the spatial encoding region matches with the sensitive region of the coil. The higher *G*_SD_, the wider sweep width. Wide sweep width means high number of points in the pulse shape, but this is not a limiting factor in modern spectrometers. Therefore, there are no significant limitations related to the spatial encoding of indirect dimension. Naturally, the maximum strength of the gradient sets the limit to the smallest observable diffusion coefficient.

The strength and length of the gradient pulses in the CPMG loop, *G*_DD_ and *G*_RD_, as well as the echo time and number of echoes are selected so that the decay of signal during the CPMG loop is sufficient and a sufficient number of echoes is observed. The stronger gradients, the faster decay. Once the *G*_DD_ and *G*_RD_ values have been set, one has to calculate a sufficient time step for collecting data points of echoes, so that the field-of-view is larger than the spatial encoding region, as well as the number of collected points, which is high enough for a reasonable spatial resolution. The length of *G*_RD_ pulse determines the maximum number of collected points. In this work, 128 complex data points were collected per each echo, which resulted in a high spatial resolution (78 μm) and 71 points in the indirect dimension of the UF DEXSY data after the removal of the extra point outside the spatial encoding region. Altogether 32 echoes were collected, which resulted in a sufficient resolution in the direct dimension. The resolution in the direct dimension can be increased by shortening the echo time and the length of *G*_RD_ pulse. This will lead to lowered maximum spatial resolution. Therefore, the maximum resolutions in the indirect and direct dimensions are coupled; if one is increased, another is decreased.

The smaller *D*, the higher *G*_RD_ is required to get sufficient diffusion decay in the CPMG loop. The higher *G*_RD_, the higher maximum spatial resolution. Therefore, the smaller *D*, the higher overall resolution in the UF DEXSY experiments, i.e., the overall maximum resolution is improved when studying more viscous systems or larger molecules or aggregates. Naturally, this requires that shortened *T*_2_ will not become a limiting factor. On the other hand, higher *D* will lead to decreased maximum resolution in UF DEXSY experiments. *D* of gases is typically about three orders of magnitude higher than that of liquids, and therefore resolution in gas phase studies may be low.

In summary, although the maximum resolutions in the indirect and direct dimensions are coupled, i.e., one cannot be increased without decreasing another, sufficient resolution can be achieved in both dimensions in typical liquid state studies. However, the resolution may be a limiting factor in gas phase studies.

## Supplementary information

Supplementary information

## Data Availability

All data are available in the main text and the Supplementary information.
