# Supplementary material for: Ultrafast diffusion exchange nuclear magnetic resonance
Source: Nat Commun. 2020 Jun 26;11:3251. doi: 10.1038/s41467-020-17079-7 (PMC7319991; doi:10.1038/s41467-020-17079-7)
Supplement: Supplementary file 1 — Supplementary information [file 41467_2020_17079_MOESM1_ESM.pdf]

Supplementary information

Ultrafast diffusion exchange nuclear magnetic resonance

Mankinen et al.

Supplementary Figures

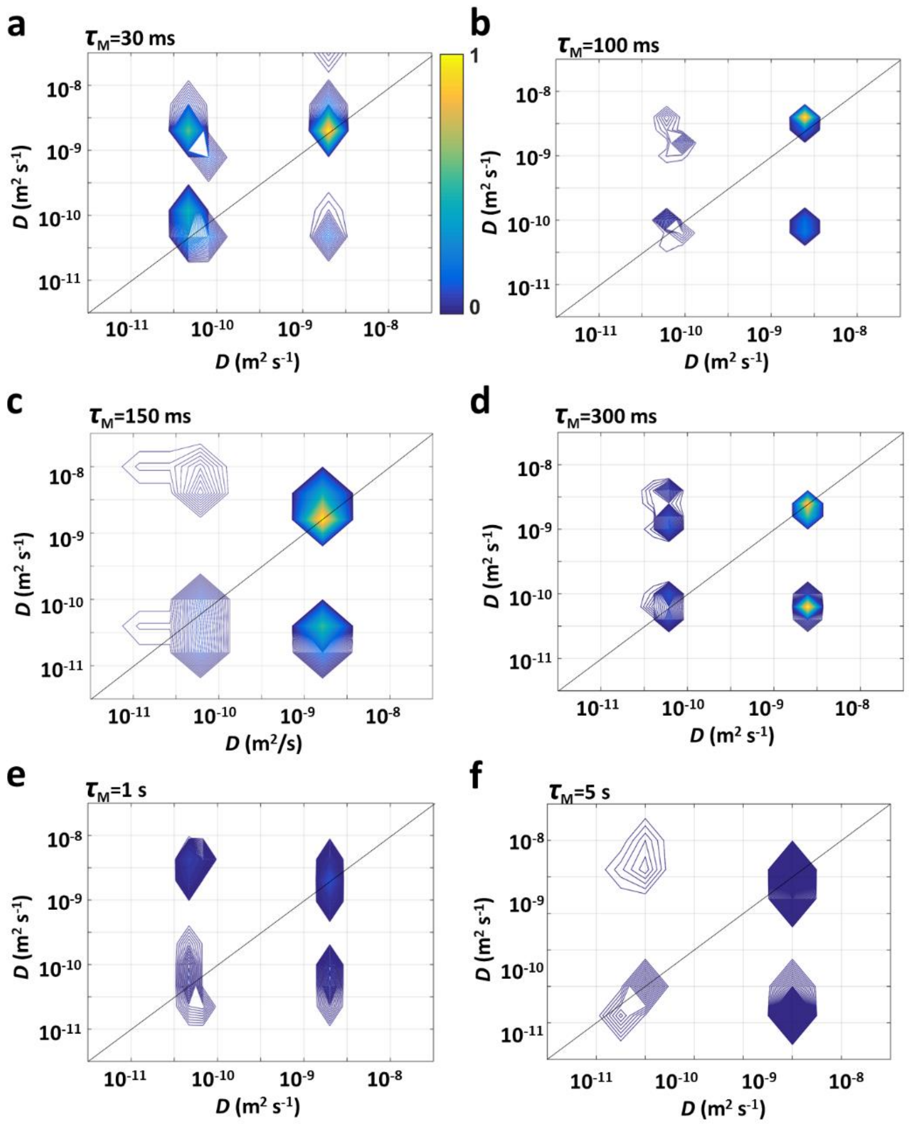

Supplementary Fig. 1 | Molecular exchange of water in aqueous sodium decanoate sample measured by the UF DEXSY. (**a-f**) DEXSY maps measured with varying mixing time τ_m.

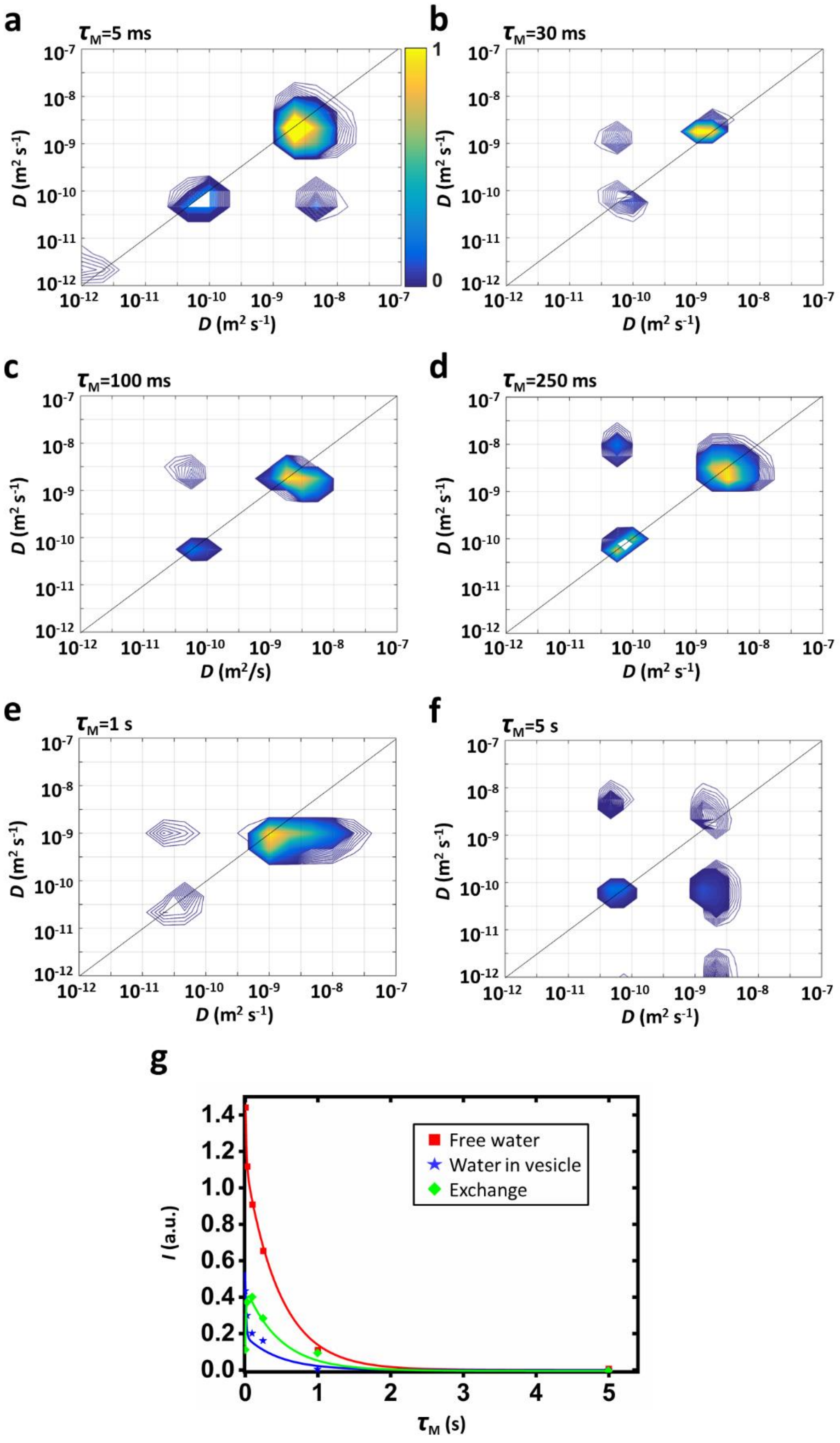

Supplementary Fig. 2 | Molecular exchange of water in the aqueous sodium decanoate sample measured by the conventional DEXSY. (**a-f**) DEXSY maps measured with varying mixing time τ_m. (**g**) The fit of the two site exchange model to the data points.

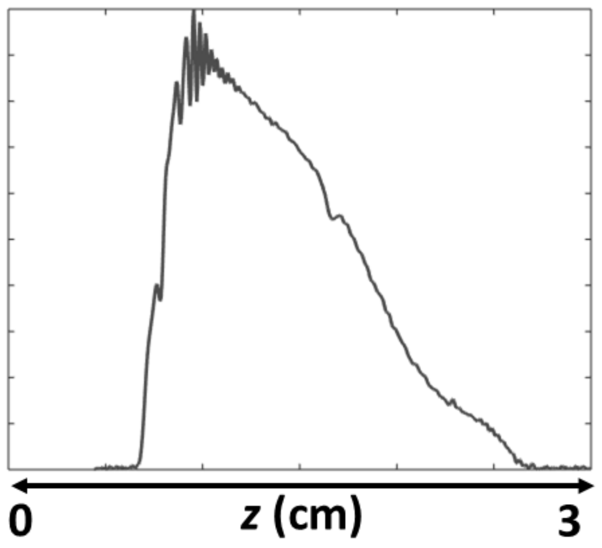

Supplementary Fig. 3 | The first row of the 2D data matrix of a doped water sample. The purpose of this figure is to demonstrate small artefacts in the UF DEXSY measurements. The left side of the spatial encoding profile includes some oscillations, because the frequency-swept pulses do not work perfectly immediately after switching them on. In the centre of the profile, there is a small dip due to switching on/off the frequency-swept $\pi/2$ pulses in the midpoint of the frequency-swept π pulses. The points suffering the artefacts can be removed from the Laplace inversion analysis.
